# Sequence Preference and Initiator Promiscuity for *De Novo* DNA Synthesis by Terminal Deoxynucleotidyl Transferase

**DOI:** 10.1021/acssynbio.1c00142

**Published:** 2021-06-22

**Authors:** Erika Schaudy, Jory Lietard, Mark M. Somoza

**Affiliations:** † Institute of Inorganic Chemistry, Faculty of Chemistry, University of Vienna, Althanstraße 14, 1090 Vienna, Austria; ‡ Chair of Food Chemistry and Molecular Sensory Science, Technical University of Munich, Lise-Meitner-Straße 34, 85354 Freising, Germany; § Leibniz-Institute for Food Systems Biology at the Technical University of Munich, Lise-Meitner-Straße 34, 85354 Freising, Germany

**Keywords:** TdT polymerase, microarray, synthetic biology, l-DNA, enzymatic DNA synthesis, photolithographic synthesis

## Abstract

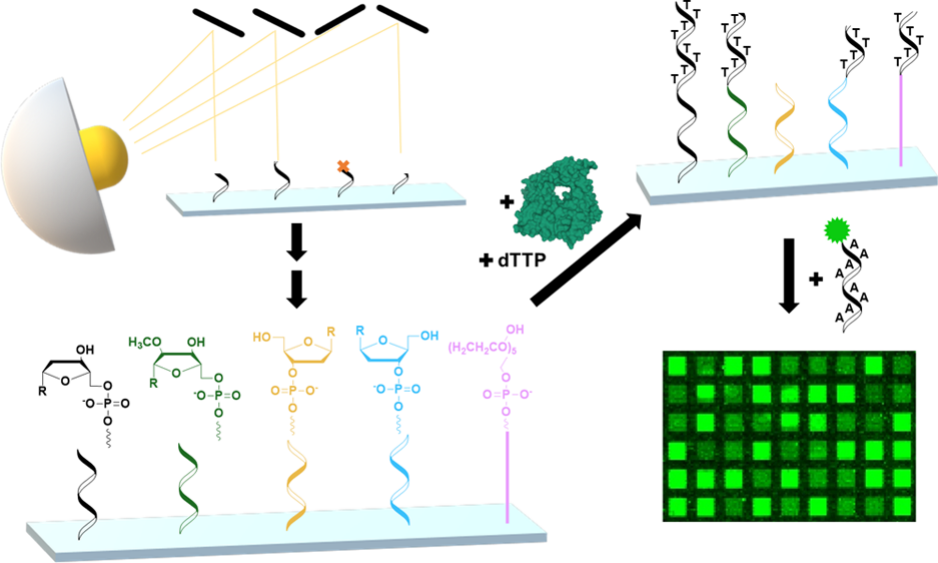

The untemplated activity of terminal deoxynucleotidyl transferase (TdT) represents its most appealing feature. Its use is well established in applications aiming for extension of a DNA initiator strand, but a more recent focus points to its potential in enzymatic *de novo* synthesis of DNA. Whereas its low substrate specificity for nucleoside triphosphates has been studied extensively, here we interrogate how the activity of TdT is modulated by the nature of the initiating strands, in particular their length, chemistry, and nucleotide composition. Investigation of full permutational libraries of mono- to pentamers of d-DNA, l-DNA, and 2′O-methyl-RNA of differing directionality immobilized to glass surfaces, and generated *via* photolithographic *in situ* synthesis, shows that the efficiency of extension strongly depends on the nucleobase sequence. We also show TdT being catalytically active on a non-nucleosidic substrate, hexaethylene glycol. These results offer new perspectives on constraints and strategies for *de novo* synthesis of DNA using TdT regarding the requirements for initiation of enzymatic generation of DNA.

Terminal deoxynucleotidyl transferase (TdT) is a member of the polX family of DNA polymerases first purified from calf thymus glands.^1,2^ In contrast to template-dependent DNA polymerases, TdT extends DNA strands at their 3′ hydroxy terminus in the presence of divalent cation cofactors^3^ and deoxynucleoside triphosphates (dNTPs), but in the absence of a template strand. This activity is of major importance in the diversification of immunoglobulins and T cell receptors in the process of V(D)J recombination of the adaptive immune system *via* random addition of nucleotides to nicked DNA strands.^4,5^ TdT’s unique ability to mediate template-independent polymerization has made it a valuable tool in a variety of molecular biology applications including finding strand breaks,^6^ modifying DNA oligomers with various NTPs,^7^ and identifying DNA damage and epigenetic modifications.^8^ Furthermore, the enzyme has proven useful for the generation of polynucleotides of high molecular weight^9^ and amphiphilic structures upon extension with BODIPY-dUTP,^10^ for detection of DNA and RNA on surfaces,^11,12^ and immobilization of DNA on solid supports.^13^ In the context of synthetic biology, template-independent DNA polymerization by TdT is, along with enzyme-based approaches,^14^ a promising alternative to chemical synthesis as many of the shortcomings of the phosphoramidite approach can be potentially avoided. In particular, coupling failures and depurination during the deblocking step limit chemical synthesis to about 200 nucleotides. The atom economy of phosphoramidite synthesis of DNA is also very poor, producing an approximately 1000-fold excess of chemical waste. Since polymerases work in aqueous solutions and are capable of fast and high-fidelity synthesis of almost arbitrary length, they promise a greener and far more efficient approach to DNA synthesis. Beyond genomics and biotechnological applications, DNA is an attractive medium for archiving digital information since it can achieve a storage density of hundreds of petabytes per gram,^15^ and data can be reliably recovered after being stored for thousands of years.^16^ Useful DNA data storage may depend on successful implementation of enzymatic synthesis since even high throughput chemical approaches are economically uncompetitive with, *e.g.*, magnetic or optical storage technologies.^17^

Several recent publications have addressed sequence control in TdT-based enzymatic synthesis. In the context of digital information storage, a looser definition of sequence control can be tolerated, allowing dNTP degradation with apyrase to limit TdT-catalyzed extension to a controlled series of short homopolymers.^18^ Precise sequence control has been achieved using photocleavable TdT-dNTP conjugates,^19,20^ 3′ photocaged dNTPs,^21^ and through the controlled release of divalent ion cofactors from photosensitive chelators.^22,23^ While it is too soon to tell which approach to sequence-controlled *de novo* DNA synthesis will be optimal, here we explore another factor critical to practical and efficient enzymatic synthesis with TdT, its initiator preferences. Experiments demonstrating TdT-based synthesis have used relatively long primer DNA oligonucleotides—20 to 60mers—as starting substrates, an impractically large number since these are made chemically and remain attached.^18,20,21,23^ In the phosphoramidite chemistry approach it is standard practice to start with one of four solid-phase columns preloaded with the first DNA nucleoside of the desired sequence. Such an approach might also be feasible in enzymatic synthesis if the initiator sequence length can be limited to one or two nucleotides, resulting respectively in 4 or 16 starting sequences or columns. This seems possible since very early research on TdT suggests a lower limit in length of the initiating DNA strand of at least 3 nt^24^ or as low as 2 nt.^25^ At the same time, we should ask whether some sequences are extended more efficiently than others, as this affects not just the initiation, but potentially each subsequent cycle of the synthesis.

A related question is whether TdT is able to extend initiator molecules other than the 3′ terminus of DNA, enabling enzymatic synthesis of chimeric nucleic acid sequences, DNA/RNA hybrids, or even conjugates where an unnatural initiator is extended with dNTPs or rNTPs. Regarding the differences in efficiency in the use of dNTPs and rNTPs, there appears to be limited ability for extension of DNA initiator strands with ribonucleotides.^26,27^ Furthermore, TdT was found to catalyze the extension of oligonucleotide strands with a variety of modified nucleoside triphosphates, for instance biotinylated,^11,28,29^ fluorescence-tagged,^30^ photo-cross-linkable^31^ or light-cleavable^21^ dNTPs and non-nucleosidic substrates,^32^ as well as fluorescent nucleobase analogues^33^ and metal base-pairs,^34^ showing rather low substrate specificity in contrast to other DNA polymerases, which could be further loosened by protein engineering efforts.^35^ An investigation of nucleoside triphosphate analogues, including arabinonucleosides and acyclic triphosphates of acyclovir and penciclovir, and their l- and d-stereoisomers showed that the stereochemistry of the triphosphates had a profound effect on substrate recognition by TdT.^36^ Whereas nucleoside triphosphate substrate specificity is rather flexible, DNA analogues in the initiating strand seem to hamper extension, for instance upon replacement of natural DNA nucleotides at the 3′ terminus with l-DNA,^37^ or when using RNA initiator strands.^38,39^

Herein, we report on the ability of TdT to extend ssDNA initiators between 1 and 5 nt in length and immobilized on a glass surface, as well as other nucleosidic and non-nucleosidic primers. Our results, which encompass the enzymatic extension of all 1364 possible sequence permutations of mono- up to pentamers for each of several nucleic acid chemistries, are based on the use of nucleic acid photolithography for the massively parallel synthesis of initiator strands on a common surface.^40^ We have recently expanded the toolbox of light-sensitive DNA phosphoramidites used in photolithographic synthesis beyond the standard 3′ → 5′ (“forward”) direction,^41^ and we are using this chemical diversity to investigate the activity of the TdT polymerase on a variety of initiators, from DNA oligonucleotides with accessible 3′ or 5′-OH groups (from “reverse” or “forward” DNA synthesis, respectively), to RNA-like nucleic acids with 2′O-methyl RNA (2′OMe-RNA), to mirror-image (l-)DNA primer strands with a terminal 5′-OH. We also examined the potential of non-nucleosidic molecules to act as initiators for TdT-mediated enzymatic synthesis by preparing polymers of hexaethylene glycol (HEG) linkers. Surprisingly, with the exception of 5′-OH d-DNA, all tested substrates were able to support some level of enzymatic extension, but with 3′ hydroxy terminated DNA clearly the optimal initiator. The extension efficiency of 3′ hydroxy terminated ssDNA by TdT is also strongly sequence dependent, with a factor of 3 efficiency difference between the best and worse pentamer initiator sequences.

## Materials and Methods

### Approach

In order to investigate the ability of TdT to extend terminal hydroxy groups of different nucleic acid chemistries, multiple replicates of each oligonucleotide strand were synthesized on the same array, each present in two versions: one where the final light-sensitive protecting group was removed at the end of the synthesis, exposing an accessible hydroxy group, whereas in the other version, the terminal hydroxy group was capped with a DMTr-dT phosphoramidite.^42^ We have previously measured the coupling efficiency of most non-RNA phosphoramidites for light-directed synthesis to be ∼99.9%, including DMTr-dT in its role as capping agent; G being the exception at 97–98%.^41,43−46^ After synthesis and deprotection, the surface-bound oligonucleotides serve as initiator sequences for dT homopolymer extension with TdT polymerase. The efficiency of polymerization was evaluated by hybridization to the extension product. Absolute fluorescent signal intensities of the capped and uncapped versions present on a single surface were compared in order to evaluate the ability of TdT to extend short oligonucleotide strands of differing chemistry and nucleotide composition. In order to allow investigation of all different monomers as initiators, and to distance the terminal hydroxy group from the glass surface, the synthesis was started with coupling of a hexaethylene glycol phosphoramidite as linker in an initial synthesis cycle.

### Photolithographic *in Situ* Synthesis

The detailed procedure for photolithographic *in situ* synthesis has already been described elsewhere.^47,48^ Briefly, microscopy glass slides (Schott NEXTERION glass D) were functionalized with a 2% *N*-(3-triethoxysilylpropyl)-4-hydroxybutyramide (95%; abcr) solution in ethanol/water/acetic acid (95:5:0.1), washed, and cured at 120 °C under a vacuum for 2 h. An Expedite 8909 nucleic acid synthesizer was used to deliver reagents for synthesis to the glass substrate. Anhydrous acetonitrile (Biosolve) and DCI activator (Sigma-Aldrich, L032000) were maintained dry under molecular sieves (Sigma-Aldrich, Z509027). The exposure solvent consisted of 1% imidazole (Sigma-Aldrich, 56750) in anhydrous DMSO (Biosolve). The oxidizer was 20 mM I_2_ in H_2_O/pyridine/THF (Sigma-Aldrich L060060). Cyanoethyl phosphoramidites were used as 0.03 M solutions in dry acetonitrile and obtained from Orgentis (5′-BzNPPOC d-DNA, 3′-BzNPPOC d-DNA), ChemGenes (5′-NPPOC l-DNA; 3′-NPPOC 2′OMe-RNA; NPPOC-hexaethylene glycol), and LINK (DMTr-dT). Phosphoramidite purity and 3′ phosphitylation selectivity was verified by ^31^P and 2D ^1^H–^31^P NMR. Coupling times varied depending on the type of phosphoramidite, between 15 s (d-DNA), 60 s (l-DNA and 2′OMe-RNA), 120 s (DMTr-dT), and 300 s (hexaethylene glycol). After synthesis, cyanoethyl and base protecting groups were removed by treating the array with ethylenediamine/ethanol (1:1) for either 2 or 15 h (3′-BzNPPOC d-DNA, NPPOC-hexaethylene glycol).

An optical system, focusing UV light from a 365 nm high-power UV-LED source (Nichia NVSU333A)^49^ onto a digital micromirror device (Texas Instruments 0.7 XGA DMD) with 1024 × 768 individually addressable micromirrors, and *via* an Offner optical relay, further onto a functionalized glass slide, allows spatially resolved removal of the photosensitive protecting groups according to a set of digital masks generated by a MATLAB program.

### Synthesis Design

Oligonucleotide microarrays used in this study are based on the same layout and design. Using the full 1024 × 768 synthesis space, a 9:25 layout (blocks of 3 × 3 synthesis pixels surrounded by 2 pixel-wide unused margins) allowed for photolithographic synthesis of 31 008 individual sequences in parallel. The full permutation library of 1 to 5 nt length was synthesized with both free and capped terminal hydroxy groups. A 25mer (“QC25”: 5′-GTCATCATCATGAACCACCCTGGTC-3′) was synthesized in parallel in order to allow for evaluation of the synthesis quality *via* a standardized hybridization. Furthermore, synthesis of T or U 18mers enabled the hybridization efficiency to be assessed during the detection of enzymatically generated dT homopolymers. All strands were grown on a single hexaethylene glycol (HEG) moiety as a non-nucleotide linker. Distribution of the sequences—and all replicates of individual sequences—on the array surface was randomized in order to compensate for any spatial effects possibly occurring upon reaction and/or hybridization. The microarrays for the investigation of non-nucleotide initiator strands were synthesized using only HEG phosphoramidites in order to obtain strands of up to nine HEG units in length, both with accessible and blocked termini.

### Extension and Detection

After removal of cyanoethyl and nucleobase protecting groups, extension reactions were performed with a mix of 0.2 u/μL calf thymus TdT (NEB M0315; 20 u/μL stock) and 100 μM dTTP (Carl Roth; 100 mM stock) in 1× TdT buffer (NEB; 50 mM potassium acetate, 20 mM tris-acetate, 10 mM magnesium acetate, pH 7.9 at 25 °C) supplemented with 0.25 mM CoCl_2_ (NEB; 2.5 mM stock) at 37 °C in a hybridization oven with rotation for 120 min in an adhesive chamber (Grace Biolabs). After incubation, the reaction mix was removed from the hybridization chamber and the array rinsed briefly by pipetting in and out nonstringent washing buffer (NSWB) (6× SSPE, 0.01% Tween-20), followed by a short wash (*ca*. 10 s) of the entire slide in final washing buffer (FWB) (0.1× SSC) and drying in a microarray centrifuge. A hybridization solution containing probe rA_18_-Cy3 (IDT; 5′-Cy3-GDDDD(rA)_18_-3′; with D being either A,G,T; 90 nM) and acetylated BSA (Promega; 0.44 mg/mL) in 1× MES buffer (100 mM MES, 1 M Na^+^, 20 mM EDTA, 0.01% Tween-20) was applied to the array surface for incubation at 4 °C without rotation for 120 min. Stringency washes were performed by washing the slide for 2 min in NSWB, 1 min in stringent washing buffer (SWB) (100 mM MES, 0.1 M Na^+^, 0.01% Tween-20) and 10 s in FWB at 4 °C. After drying, the slides were scanned at 532 nm at a resolution of 5 μm using a GenePix Personal 4100A scanner.

### Data Analysis

The Cy3 fluorescent signal intensities observed upon hybridization to the enzymatically generated homopolymer served as a measure of successful extension of initiator strands. Alignment of the scans with the underlying design using NimbleScan 2.1.68 (NimbleGen) allowed for data extraction for each individual feature. The data were analyzed using Microsoft Excel. Fluorescent signal intensities observed on features with blocked termini were treated as background noise and subtracted from the signal measured for the version with an accessible terminal hydroxy group. Sequence logos were created using WebLogo (weblogo.berkeley.edu).^50^

## Results and Discussion

**Figure 1:**
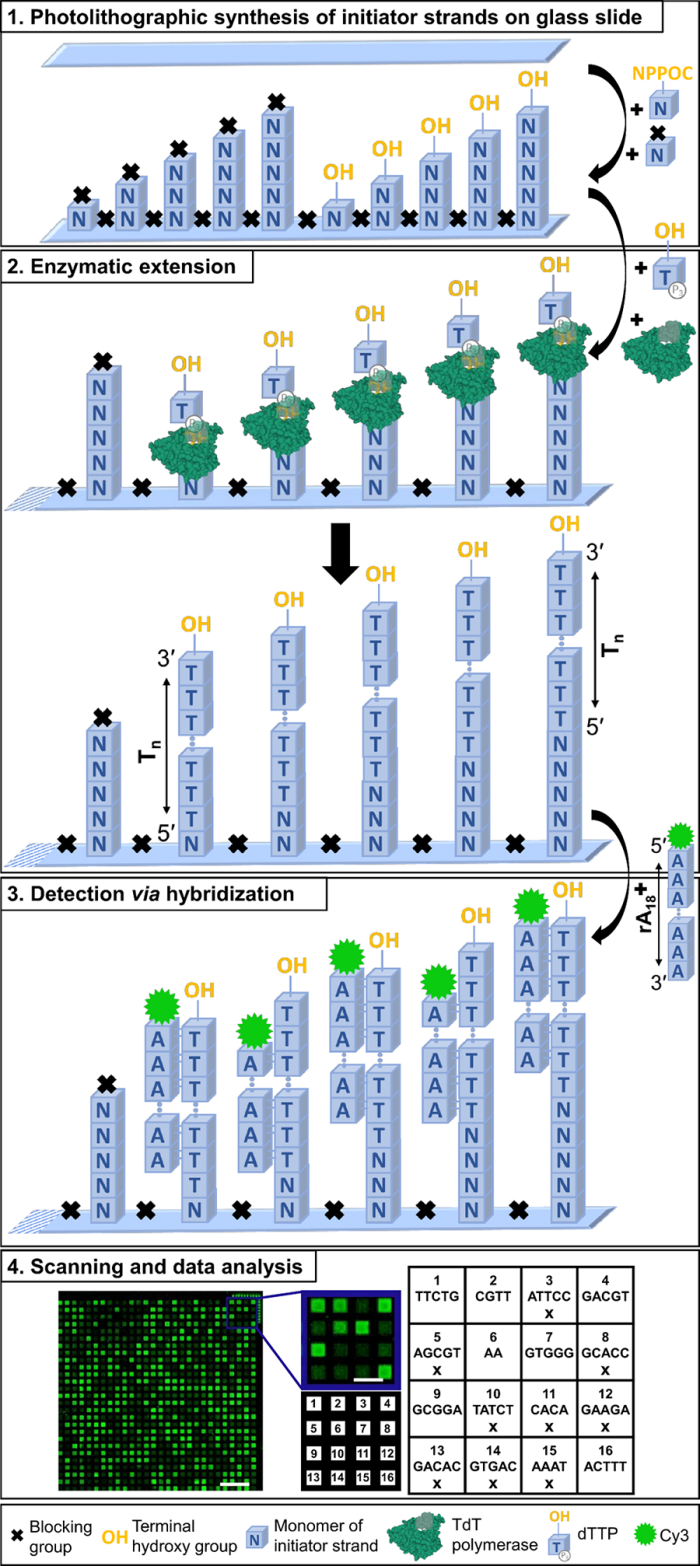
Schematic representation of the experimental design and assays. (1) Two variants of all possible permutations of mono- to pentamers, either with accessible terminal hydroxy group (OH) or with DMTr-blocked terminus (×), were synthesized on a glass slide *via* photolithography. (2) The immobilized initiator strands were then extended enzymatically by TdT using dTTP as substrate, generating dT homopolymers. (3) Poly dT strands were detected *via* hybridization with a Cy3 labeled complementary probe. (4) Scanning of the microarray allows for fluorescent signal intensities at different positions to be assigned to specific sequences. The scan to the left corresponds to 2.4% of the total synthesis area (scale bar 300 μm). In more detail, the close-up of 16 features (scale bar 100 μm) and the corresponding layout beneath are shown with a grid next to it, indicating the sequences synthesized at specific positions. Features with blocked termini (×) exhibit much lower fluorescence signal intensity than those with strands accessible for extension. TdT model adapted from PDB: 1JMS.

The ability of TdT to extend all possible mono- to pentamers of nucleotide chains with differing sugar chemistries was investigated *via* hybridization to the product of extension. This setup allowed not only for a comparison of the extension efficiency of different chemistries, but also for the identification of preferences in nucleotide composition as well as the minimal length still allowing for enzymatic polymerization. Besides nucleic acid pentamers, we also prepared polymers of hexaethylene glycol (HEG) containing up to nine units. Due to the uncontrolled mode of action of TdT randomly adding nucleoside triphosphates to the growing chain, we restricted our study to only dTTP as a substrate in order to generate poly dT strands detectable in a hybridization-based assay with a fluorescently labeled complementary rA_18_ probe, as shown in Figure 1. Synthesis only using a single type of dNTP allows us to isolate the impact of the initiating sequence on extension efficiency from biases in the incorporation of dNTPs that have been observed *in vivo*(51) and *in vitro*.^19,24^

**Figure 2:**
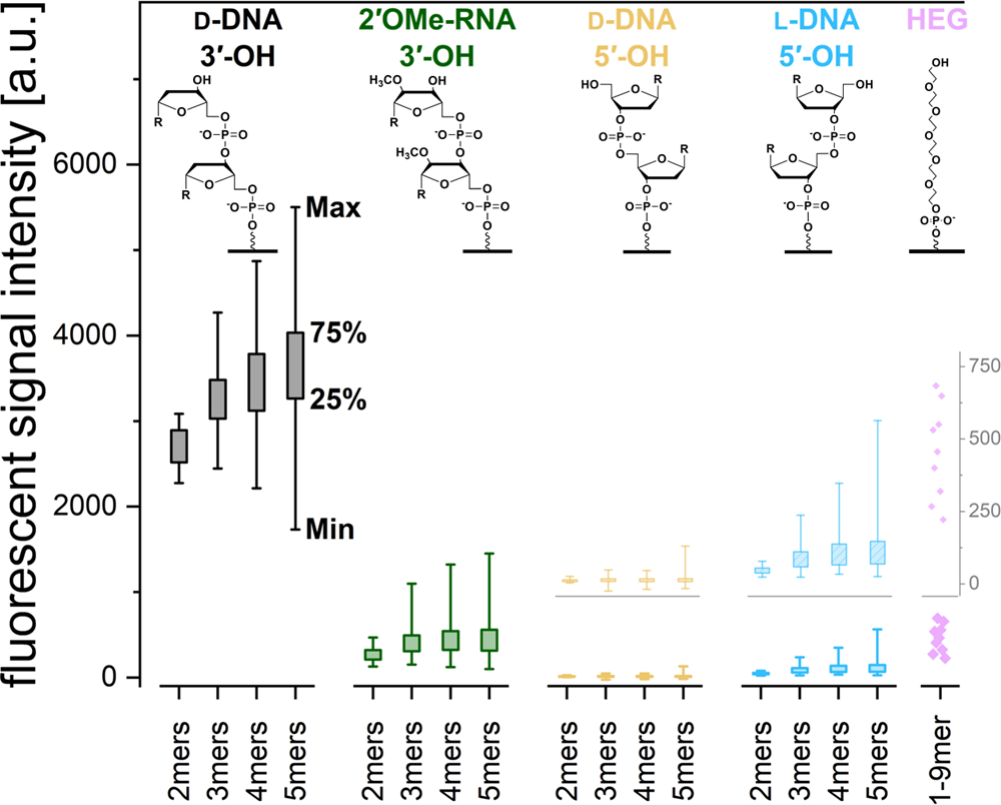
Fluorescent signal intensities after background subtraction for five different types of initiating strands. The structure of the corresponding dimer (monomer for HEG), immobilized to the surface is illustrated. For each type and length of oligonucleotide strands, the 0th, 25th, 75th, and 100th intensity percentiles are shown, based on all possible 4^*n*^ data points for each initiator strand of length *n*. Hexaethylene glycol *n*-mers are plotted with dots. The greyed-out insert for 5′-OH d-DNA, 5′-OH l-DNA, and HEG shows this lower range of signal intensity in more detail.

The analysis of fluorescent signal intensities measured upon hybridization to the enzymatic reaction products allowed for extension efficiencies to be compared. Figure 2 shows the range of signal intensities observed for the extension of initiators of differing nucleic acid chemistries, with lowest and highest fluorescent signal intensities detected and after background subtraction (sequences with blocked terminal hydroxy group). We set the threshold to evaluate TdT’s general ability to extend an initiator as the average of signal intensities for blocked sequences plus three times their standard deviation.

### d-DNA 3′-OH Extension

With the cognate substrate of TdT being single-stranded DNA with a 3′-OH terminus, we expected the highest extension efficiency for this substrate. Indeed, signal intensities plotted in Figure 2 clearly show 3′-terminated d-DNA as the favored substrate of all five different chemistries. Focusing on the left panel of Figure 2, the extension reaction efficiency increases with the length of the initiating strands. However, there is also a clear dependence on the nucleotide composition, to the extent that some sequences of longer oligonucleotides can be less efficient initiators than the shortest.

**Figure 3:**
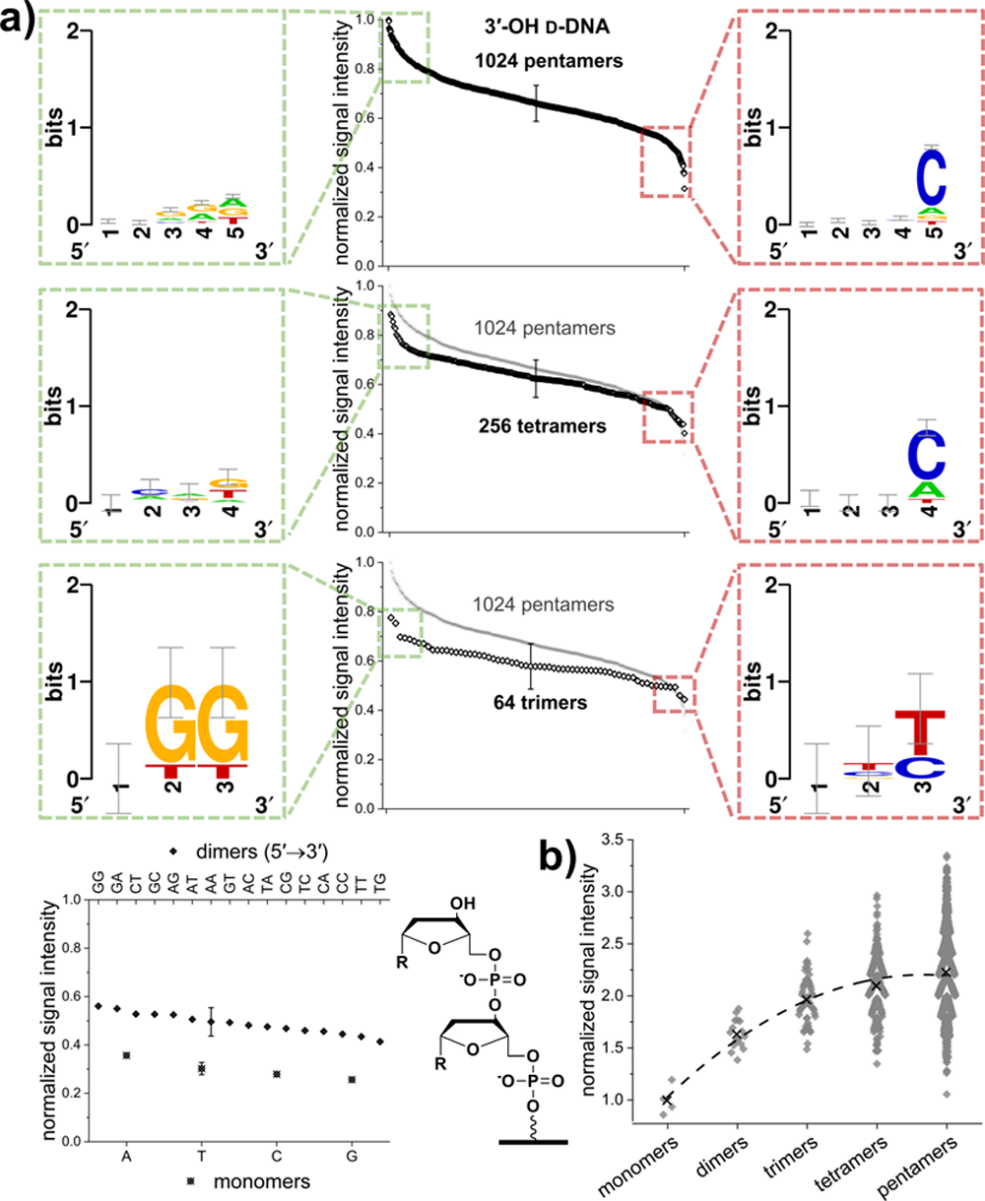
Analysis of extension of 3′-OH d-DNA initiating strands. (a) Fluorescent signal intensities were normalized to the maximum and clustered according to length, with representative SEM error bars. Panels to the left illustrate sequence patterns (5′ → 3′ direction) from the data for the 10% highest signal intensities framed in green, whereas panels to the right show the data for the 10% lowest signal intensities for penta-, tetra-, and trimers (top to bottom) framed in red. Data for pentamers are repeated in gray in the subsequent plots for comparison. For monomers and dimers, data are plotted from highest to lowest signal intensity with the corresponding sequence specified by the labeling of the top and bottom *x*-axis for dimers and monomers, respectively. Next to this plot, the chemical structure of a dimer immobilized to the glass surface serves as a guide for straightforward identification of differences between the chemical variants tested for initiation in this and subsequent figures. (b) Fluorescent signal intensities normalized to the average of all monomers and clustered according to strand length. Averages for each strand length are indicated by an “×”. The dotted second order polynomial fit through the averages serves as a visual guide.

Investigation of the full permutation library allowed us to identify which nucleotide sequences are preferentially extended. Figure 3a provides an overview of the trends of initiating sequences yielding the 10% highest (left, framed in green) and lowest (right panel, framed in red) extension efficiency. Consensus sequence logos illustrate these trends. While the nucleobase sequence is less relevant for mono- and dimers, for tetra- and pentamers extension is least efficient in the case of a deoxycytidine in the 3′ terminal position, with the lowest signal detected for the sequences TAGAC and GATC (all sequences 5′ → 3′). In the case of trimers, the two isolated data points at the top end of the range correspond to the sequences GGG and CGG, emphasizing the preference for G in the terminal positions of efficient initiators of this length. The two data points at the low end of the sigmoidal curve represent results for ACC and TGC, with the corresponding consensus sequence again clearly showing that a terminal C is not favored for extension. Investigation of the effect of strand length is shown in Figure 3b, where the average signal intensity of all sequences of a specific length are normalized to the average signal intensity of monomers. Independent of nucleotide composition, the results show that the efficiency of initiation increases with strand length, with pentamers facilitating—on average—2.2× higher initiation efficiency compared to monomers. Applying a second order polynomial fit as guide suggests elongation efficiency asymptotically approaches a maximum, hinting that increasing initiating strand length further may not significantly improve average efficiency. Still, the wide range of signal intensities detected for each length emphasizes even more the impact of nucleotide composition.

### 2′OMe-RNA 3′-OH Extension

**Figure 4:**
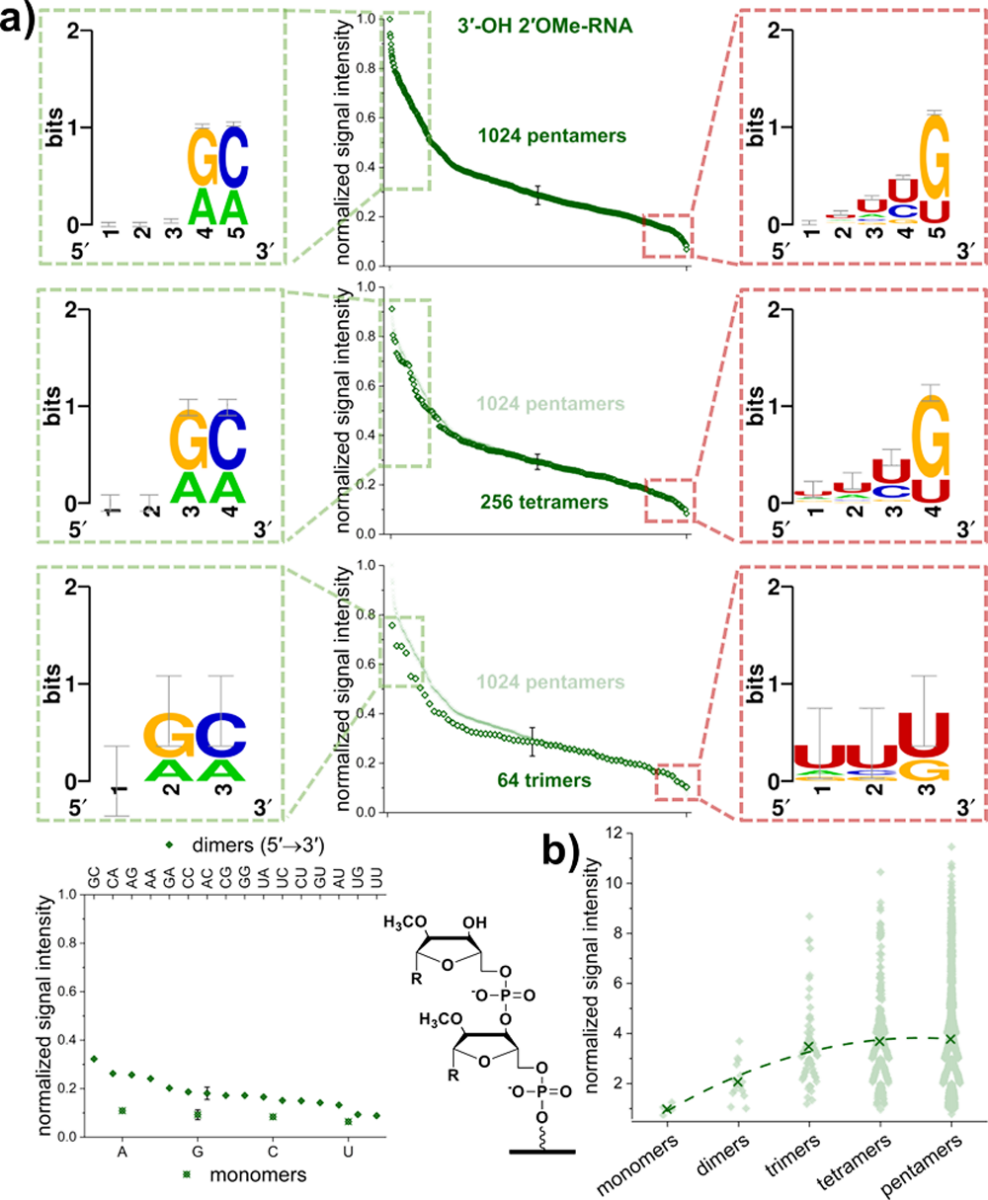
Extension analysis of 2′OMe-RNA initiating strands with terminal 3′-OH. (a) Fluorescent signal intensities were normalized to the maximum signal detected and clustered according to their length, with representative error bars corresponding to 2× SEM for better visibility. Panels to the left illustrate sequence patterns (5′ → 3′) emerging from the data for the 10% highest signal intensities (framed in green), whereas panels to the right show the data for the 10% lowest signal intensities framed in red. Data for pentamers are also shown in the following plots for comparison, pointing to the similarity in shape between graphs for differing strand lengths. For monomers and dimers, data are plotted from highest to lowest signal intensity with the corresponding sequence specified on the labeling of the top and bottom *x*-axis for dimers and monomers, respectively. Next to this plot, the chemical structure of a dimer immobilized to the glass surface serves as a guide for identification of differences between the chemical variants tested for initiation. (b) Fluorescent signal intensities normalized to the average of all monomers and clustered according to strand length. Averages for each strand length are indicated by “×”. A polynomial fit through the averages serves as a visual guide.

Investigation of enzymatic extension of short strands of 2′OMe-RNA with a terminal 3′ hydroxy group synthesized on the surface clearly shows that TdT is able to use it as a substrate, albeit at lower efficiency than its d-DNA counterpart. In comparison to 3′-OH d-DNA, TdT exhibits distinct preferences for sequence composition in 2′OMe-RNA initiator strands, as shown in Figure 4a. Indeed, the nucleobase in the terminal position of the strand and the adjacent one have a major impact on the efficiency of extension, with adenine and cytidine nucleotides being favored in the terminal position when next to adenine or guanosine nucleotides. In contrast, both guanosine and uracil nucleotides at the 3′ terminus have a negative impact on the efficiency of strand extension. Of note, we found that extension of a 2′OMe uracil nucleotide is disfavored in almost all cases, including for mono- and dinucleotides. Comparison of the efficiency of initiation based on strand length shows a significant leap from monomers to pentamers (Figure 4b). The second order polynomial fit to the average signal of all sequences of a specific length levels off for tetramers and pentamers, suggesting a close-to-maximum efficiency already for initiating strands with five nucleotides in length. In comparison to the d-DNA (3′-OH) substrate, the position of the average signal intensity relative to the range of signal observed for longer initiators is striking. The distribution of data points clearly indicates that most of the sequences are being extended with low efficiency, keeping the average efficiency of initiation of pentamers at a level of approximately 4× compared to monomers, whereas some outstanding variants even show initiating efficiencies of more than 10× that of monomers.

### d-DNA 5′-OH Extension

In order to investigate if 5′-OH DNA extension is possible, the TdT reaction mix was applied to a microarray populated only with d-DNA tethered to the surface at the 3′ end and with a terminal 5′-OH. In this case, only very low fluorescent signal intensities were detected (see Figure 2). The average values for all sequence permutations and for lengths between monomers and pentamers were below the limits of detection (determined using the data for DMTr-capped strands as unextendable controls), indicating that strands of d-DNA with terminal 5′ hydroxy group are not suitable substrates for extension with TdT.

### l-DNA 5′-OH Extension

**Figure 5:**
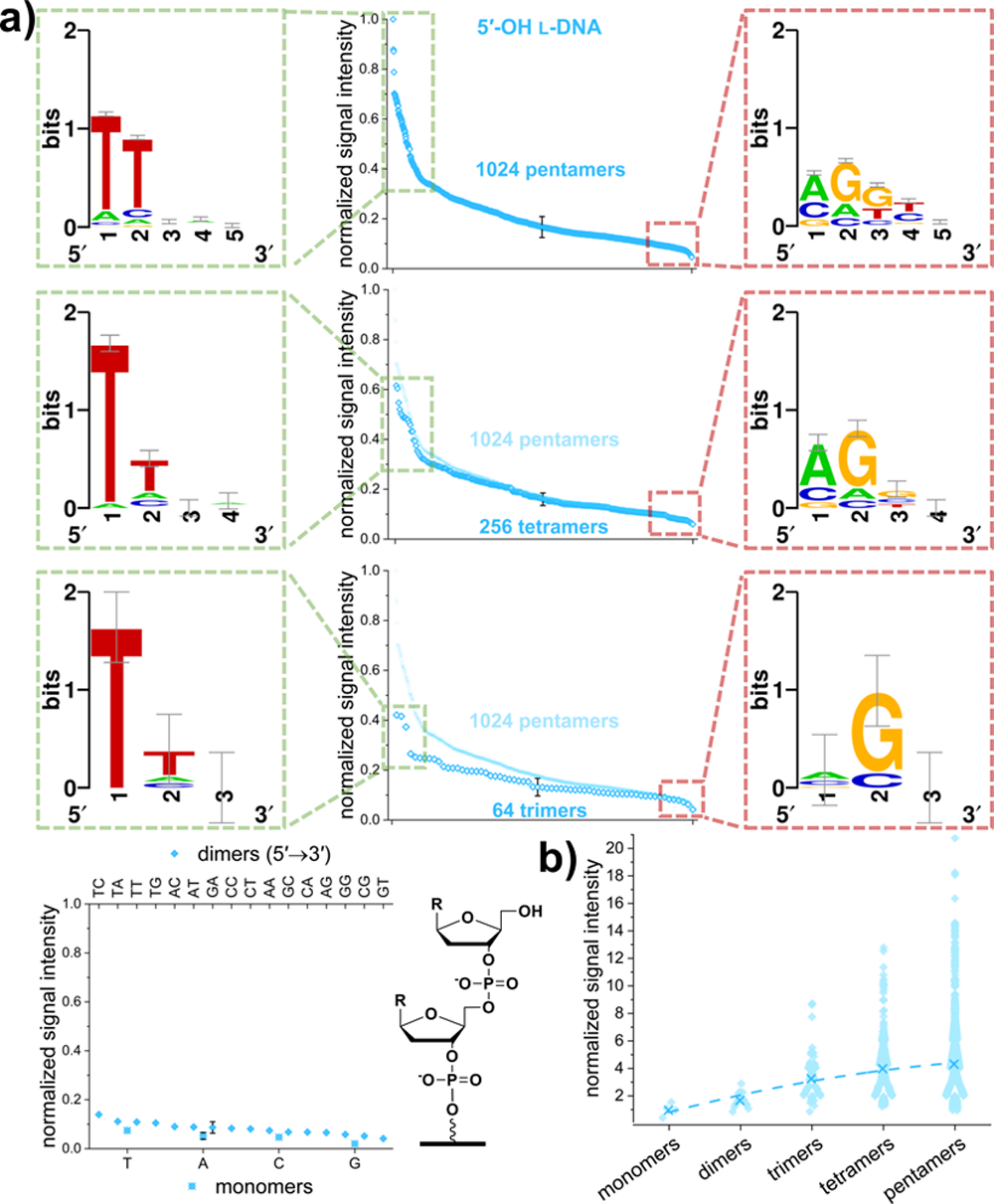
Analysis of 5′-OH l-DNA strand extension data. (a) Fluorescent signal intensities were normalized to the maximum and grouped by length, with representative error bars corresponding to 2× SEM for better visibility. Panels framed in green illustrate sequence patterns for the 10% highest signal intensities, whereas panels framed in red show the data for the 10% lowest. Pentamer data are repeated in subsequent plots for comparison, pointing to the similarity in shape between graphs for differing strand lengths. For monomers and dimers, data are plotted from highest to lowest signal intensity with the corresponding sequence specified on the labeling of the top and bottom *x*-axis for dimers and monomers, respectively. Next to this plot, the chemical structure of a dimer on the glass surface serves as a guide for identification of differences between the chemical variants tested for initiation. (b) Fluorescent signal intensities normalized to the average of all monomers and clustered according to strand length. Averages for each strand length are indicated by “×”. The dotted line is a second order polynomial fit through the averages.

Our recent report establishing photolithographic *in situ* synthesis for mirror-image DNA (l-DNA)^46^ motivated us to investigate the activity of TdT on this non-natural substrate. Surprisingly, we indeed were able to detect significant extension, albeit lower than for 3′-OH terminated d-DNA (Figure 2). The sigmoidal curves generated in order to show the distribution of fluorescent signal intensities among all sequence permutations of equal length in Figure 5a cover a considerable range, indicating that the sequence of the initiating strand has a critical impact on the efficiency of extension. Analysis of nucleotide composition of the l-DNA initiator strands unambiguously shows a strong preference for l-dT at both the 5′-OH terminus and at the adjacent position for TdT extension for all initiator lengths investigated. In contrast, the identities of nucleotides more distant from the site of extension are mostly irrelevant. Interestingly, poorly extended substrates fall into the same range of low fluorescence regardless of primer length, as indicated by overlapping the greyed-out curve for pentamers with data points of tetramers and trimers. Whereas short strands do not allow for considerable extension, with signal intensities for monomers on average being hardly above the limit of detection, thymine is the favored nucleobase even in this context. Exceptionally high signal intensities compared to other sequences of the same length were measured for the pentamers TTAAA, TTAAG, and TTAAT, the tetramers TTAA and TTAT, and the trimers TTT, TTC, and TTA (all 5′ → 3′), as illustrated by their prominent positions as individually discernible data points at maximum signal intensity. Comparing the average signal intensities for each initiator length with one another in Figure 5b once again emphasizes the significant increase in the efficiency of initiation with strand length. A second order polynomial fit to the average serves as a visual guide and suggests a maximum efficiency of initiation for strands approximately five nucleotides in length. On average, signal intensities for pentamers are 4.3× higher than for monomers. However, a few isolated data points at the top end of the range show that the efficiency of initiation is strongly influenced by the l-DNA sequence, as initiating efficiencies for individual pentamers can be up to 20× higher than for the monomer average.

### Hexaethylene Glycol Extension

**Figure 6:**
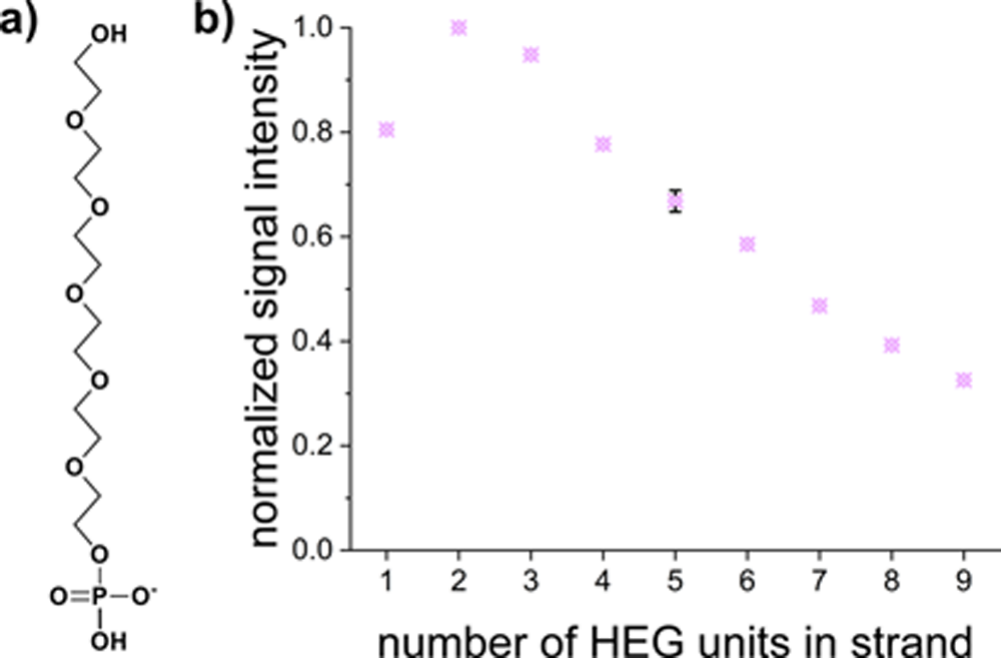
Extension of hexaethylene glycol strands. (a) Molecular structure of a single HEG unit. (b) Fluorescent signal intensities, normalized to maximum signal detected for dimers, show a decreasing trend with increasing number of HEG units in the initiating strand (error bar representative for SEM).

In order to assess the ability of TdT to act on primary hydroxy groups of non-nucleosidic substrates, microarrays with strands of hexaethylene glycol (molecular structure shown in Figure 6a), ranging from one to nine units in length, were synthesized. Surprisingly, these initiator strands were extended by the enzyme, with fluorescence signals clearly above the LOD and in the same range as for 2′OMe-RNA and l-DNA (Figure 2). Investigating the dependence of fluorescent signal intensities as a function of initiating strand length hints at shorter strands being extended more efficiently than longer ones (Figure 6b).

DNA extension with TdT has been studied for over 60 years, but surprisingly few specific details have been established regarding the initiator preferences of this unique polymerase. Particularly in the context of *de novo* DNA synthesis, these preferences are crucial to developing a practical and efficient approach competitive with phosphoramidite chemistry. Recent efforts in this field have used initiator strands 20 to 60 nt in length and of heterogeneous nucleobase composition. Although earlier research has shown that short TdT initiators are also functional, a lack of information on the initiator length dependence for TdT polymerization efficiency may have contributed to the choice of very long initiators. The crystal structure of murine TdT indicates that only three nucleotides at the 3′-hydroxy end are ordered within the polymerase, whereas additional ones are outside the polymerase and disordered.^52^ This along with the 3 nt minimum initiator length indicated by Kato *et al.*(24) suggests that any benefit to longer initiators would be due to more indirect mechanisms such as 1D diffusion along the strand facilitating the localization of TdT to the 3′-hydroxy end. While 1D diffusion along DNA has been identified for the T7 RNA polymerase,^53^ there is no evidence of a similar process for DNA polymerases in the absence of accessory sliding clamp factors.^54^ Although our data only extends to pentamers, it clearly shows that initiators longer than about 5 nt are unlikely to significantly enhance TdT polymerization efficiency. This is true for TdT’s natural substrate, 3′-hydroxy terminated DNA, for which we observe polymerization efficiency flattening beyond an initiator length of 4 nt (Figure 3b), as well as for the non-natural substrates 2′OMe-RNA and 5′-hydroxy terminated l-DNA (Figures 4b and 5b). On the short end of initiator length, we were able to observe significant polymerization for both monomers and dimers. This observation stands in contrast to the 3 nt lower limit of Kato *et al.* However, in our experiments these short DNA strands are linked to relatively long hexaethylene glycol strands, which themselves can function as initiator strands.

For 2′OMe-RNA, lower efficiencies of TdT extension were expected considering earlier reports of RNA primers not being extended, neither with dNTPs nor with rNTPs.^38^ Extension of a DNA primer with rNTPs showed an upper limit of 3–4 added nucleotides,^26^ leading to the hypothesis that the enzyme stops extension as soon as the initiator strand transitions from DNA to RNA. Comparing these reports with our own results, we observe that methylated RNA analogues can indeed be extended. Since a minimum extension length of seven dT nucleotides are necessary to provide a detectable hybridization signal with the rA_18_-Cy3 probe, our data show that the initiating strand must have been extended by at least seven dT nucleotides. Considering the additional steric hindrance from the 2′-methyl compared to unmodified RNA, the extension of 2′OMe-RNA with bulkier methyl groups suggests a more complex gating mechanism. Since 2′OMe-RNA, HEG, and l-DNA are functional, albeit inefficient initiating strands, whereas 5′-OH extension of d-DNA does not occur, the results suggest that TdT has evolved to exclude this last substrate rather than to be highly specific for 3′-OH DNA extension.

Regarding the activity of TdT on mirror-image DNA substrates, only a few reports exist. Already in 1995, Focher *et al.*(55) demonstrated the ability of calf thymus terminal transferase to extend a dT_20_ primer of d-DNA (with a blocked 5′ terminus) upon addition of l-dTTP. However, extension stopped after 1–2 nt, indicating that this short stretch of l-DNA with a terminal 3′-OH is not a functional initiator. Another study on the extension of a d-DNA primer with a single l-dT incorporation at the 3′ end showed the extension using d-dNTPs is aborted after 1–2 nt. The authors speculated that a distortion of orientation initiated by presence of the l-nucleotide could result in termination of extension.^37^ However, all these investigations focus on extension of oligonucleotides with a terminal 3′ hydroxy group in solution. In contrast, the present study used l-DNA phosphoramidites in 3′ → 5′ synthesis direction using pure 5′-NPPOC 3′-L phosphoramidites, resulting in strands immobilized to the surface and with an accessible terminal 5′ hydroxy group. In this context, comparing the results with those for the corresponding 5′-OH d-DNA initiator strands is especially surprising. As shown in the inset in Figure 2, signal intensities for hybridization after applying TdT to l-DNA initiator strands were significantly higher relative to the corresponding 5′-OH d-DNA initiators, which were simply not extended at all, also indicating the absence of d-DNA contamination in the l-DNA building blocks. We surmise that the structural differences between d- and l-DNA play a role in the mirror-image form acting as a potential substrate. The left-handed conformation of l-DNA prevents not only hybridization to d-DNA, but also interaction with l-enzymes in the active center.^56,57^ Since the structure of d-DNA oligonucleotides with a 5′ terminal hydroxy group did not prove suitable as a substrate for extension, the conformational change to its mirror-image pendant seems to represent the variation required to fit the active center of the polymerase and allow for strand extension, albeit with much lower efficiency than at the 3′-OH of d-DNA substrates. Interaction of mirror-image DNA oligonucleotides with a natural DNA polymerase has been reported recently, however, with a substantial difference in location of the binding site compared to d-DNA.^58^ To the best of our knowledge, this is the first report of a native DNA polymerase in l-conformation showing cross-chiral activity *via* catalysis of a reaction on a mirror-image DNA substrate, thereby generating chimeric l-/d-DNA strands. TdT was found to preferentially extend l-DNA strands featuring a thymidine residue at the 5′ terminus, and efficiency of initiation was enhanced considerably compared to extension of monomers by increasing the length of strands with one or more terminal T nucleotides. Given the enhanced intracellular stability of mirror-image oligonucleotides,^59^ their potential as drug delivery vehicles in the form of micelles generated *via* TdT-mediated extension of l-DNA aptamers is an alluring prospect.^10^

That TdT can elongate even short initiator sequences is of major importance for enzymatic *de novo* synthesis of DNA since any initiator must be either removed after synthesis or chosen to match the 5′ end of the desired sequence. Presumably, any initiator must be synthesized chemically, negating many benefits of enzymatic synthesis, at least for longer initiators. Fortunately, short initiators work reasonably well, such that in the manner of current solid phase synthesis of DNA, synthesis columns preloaded with the first 5′ nucleoside on a long and cleavable linker could be used. The elongation efficiency of monomers is about a third of that of pentamers, thus requiring longer initial cycles until a more optimal length is reached. The extension of non-natural initiators such as HEG and l-DNA by TdT could also be used as a workaround; even retained as a 5′ extension to the desired DNA sequence, these initiators are largely bio-orthogonal and would not interfere in many downstream applications, or could potentially be selectively removed chemically or enzymatically after synthesis. Nevertheless, the demonstrated success of nucleotide monomer initiators for *de novo* TdT synthesis seems more useful in most contexts. The use of alternative initiator chemistries still supports the possibility to use TdT to create mixed nucleic acid chimeras, particularly since several non-DNA nucleoside triphosphates have been found to be accepted by TdT.^11,27,28,30−32^

The strong sequence dependence of TdT initiator extension is a potential complication in TdT-based *de novo* synthesis. Crystallographic studies of murine TdT indicate that three consecutive nucleotides are at well-defined positions within the polymerase,^52^ suggesting that TdT processivity is potentially sensitive to the identity of the last three bases, but unlikely to be significantly affected by further upstream bases. This hypothesis is largely confirmed by our data. Consensus logos for the 3′-hydroxy DNA initiators extended most efficiently by TdT include only the last three 3′ bases for both the pentamers and the tetramers, and the last two 3′ bases in the case of the trimers (Figure 3a). In the case of the most poorly extended initiators, a consensus only appears for the terminal 3′ base for the pentamers and tetramers, whereas there is a small contribution from the second nucleobase in the case of the trimers. In the case of the 2′O-methyl-RNA and l-DNA initiating strands, again only two or three bases adjacent to the 3′-hydroxy end contribute significantly, either positively or negatively, to the polymerase extension efficiency. For extension of TdT’s natural substrate, we found a 3-fold range in efficiency between the best and worse initiator sequences for pentamers, with the worst sequences resulting in polymerization yields similar to the average values obtained for monomer extension, about 2.5-fold lower than the average for pentamers. Poorly extended pentamers are characterized by a 3′ cytosine, whereas the more optimal initiators are less well-defined but are generally missing cytosines in the two terminal positions. Very similar trends are apparent for tetramers, and for trimers the pattern is less well-defined, but guanines in the first two 3′ positions and cytosine or thymine at the 3′ are correlated with best and worse extension, respectively. For monomers and dimers, the reduced number of possible initiators and the smaller range between the best and worse initiators prevents a similar sequence assessment.

In the case of 2′OMe-RNA initiating strands, we measured a ∼12-fold range in initiator extension efficiency between the best and worst pentamer sequences. For tetramers, trimers and dimers, the range decreases with length but is far larger than for 3′-hydroxy d-DNA initiators of the same length (Figure 4). Only in the case of the monomers is the efficiency largely independent of nucleobase identity. This strong sequence dependence results in well-defined consensus sequence logos. The nucleobases immediately adjacent to the 3′ terminus are consistently cytosines and adenosines for the best initiators and guanines and uracils for the worst initiators. That the sequence dependence for 2′OMe-RNA is completely different from that of d-DNA is not surprising given that the methoxy group must substantially alter the conformation of the initiator within TdT, such that, apparently, only sequences with the rather specific pattern revealed by the consensus logos are able to function as a substrate for polymerization.

As for 2′OMe-RNA, TdT is also able to extend the 5′ hydroxy of l-DNA with low but clearly measurable yield. Similarly, the sequence-dependent range of extension efficiency is very large, about 20-fold, and associated with specific sequence patterns. Better initiators share a pair of terminal thymines, whereas the worse initiators omit this base in these positions and instead favor adenine and guanine. Since this substrate is the wrong end of the enantiomorph of the natural substrate of TdT, it appears that the polymerase is rather unspecific and will add nucleotides to many hydroxy-bearing molecules that fit within its binding site. This hypothesis is supported by the extension of hexaethylene glycol, which has little resemblance to single-stranded DNA other than flexibility and a terminal hydroxy group. Figure 6 clearly indicates fluorescent signal intensity, corresponding to extension efficiency, reaching a maximum for two linked HEG molecules. For the cases of both one and two HEG units, the extension efficiency is greater than for any of the substrates except the natural DNA substrate and the ∼10% best 2′OMe-RNA initiators. We attribute the loss of efficiency with further extension to the primary alcohol becoming less accessible within a polyethylene glycol tangle.

By comparing absolute signal intensities (Figure 2) for the different chemistries and averaged values across all sequence variations (summarized in Table 1), d-DNA with available 3′-OH represents the most efficient polymerization initiator. The data shown in Table 1 indicate that the extension of even the poorest initiator sequence made of 3′-OH d-DNA remains a better primer than any other type of substrate. These differences should be taken into account when considering nonstandard initiators. In such cases, the reaction conditions should be adapted, with for instance longer reaction times or an increase of TdT concentration.

**Table 1:** Results Summary Regarding Sequence and Length Dependence of Initiation Efficiency on Oligonucleotide Extension with TdT Polymerase and dTTP for Various Types of Initiator Chemistries^a^

		sequence motifs (5′→3′) for 10% highest/lowest signal					most/least efficiently extended substrate	
	normalized average signal^b^		dimers	trimers	tetramers	pentamers	sequence	corresponding normalized signal^d^
d-DNA 3′-OH	0.652	highest	G _	_ G G	_ _ _ _	_ _ _ _ _^c^	TTCAT	1.000
		lowest	T _	_ _ T/C	_ _ _ C	_ _ _ _ C	TAGAC	0.315
2′OMe-RNA 3′-OH	0.086	highest	_ _	_ G/A C/A	_ _ G/A C/A	_ _ _ G/A C/A^c^	GGUGC	0.264
		lowest	U _	U U U/G	_ _ U G/U	_ _ U U G/U	UGUUG	0.018
l-DNA 5′-OH	0.021	highest	T _	T T _	T T _ _	T T _ _ _^c^	TTAAA	0.102
		lowest	_ _	_ G _	A G _ _	A G G _ _	AGT	0.004
HEG	0.083	n.s.	n.s.	n.s.	n.s.	n.s.	(HEG)_2_	0.125
d-DNA 5′-OH	0.002	n.e.	n.e.	n.e.	n.e.	n.e.	n.e.	n.e.

a “_”, no distinct nucleotide occurring at higher frequency at this position; n.s., no sequence dependence; n.e., no extension.

b Fluorescent signal intensities averaged over all lengths and sequences, then normalized to highest signal intensity (1 = d-DNA 3′-OH “TTCAT”).

c Initiator length showing highest fluorescent signal for extension.

d Fluorescent signal intensity of best or worst sequence for initiation, respectively, normalized to highest signal intensity.

## Conclusions

Our study brings important new information to the activity spectrum of TdT polymerase. In addition to its already well-described broad range of acceptance for different types of modified (d)NTPs and their analogues, we show here its ability to extend other types of initiators as well. Although the natural substrate of TdT, the 3′ terminus of DNA, clearly outperforms 2′OMe-RNA (3′-OH), l-DNA (5′-OH), and hexaethylene glycol in enzymatic extension efficiency, that these initiators are extended at all is remarkable. With the investigation of sequence dependence on the efficiency of extension, and the detection of initiation of extension even for single nucleotides, our results open up new opportunities for decoupling approaches for enzymatic *de novo* synthesis from chemical synthesis of DNA and illustrate substrate diversity coexisting with sequence specificity for the template-independent TdT polymerase.

## Supporting Information Available

Description of SI contents (PDF)
Data S1: Fluorescent intensity data of all experimental data in spreadsheet format (XLSX)
Data S2: Layout design file with the location and identity of all probes for the HEG arrays (TXT)
Data S3: Layout design file with the location and identity of all probes for the oligonucleotide arrays (TXT)
Data S4: High resolution fluorescent scan data for the 2′OMe-RNA data (TIF)
Data S5: High resolution fluorescent scan data for the d-DNA 3′-OH extension data (TIF)
Data S6: High resolution fluorescent scan data for the d-DNA 5′-OH extension data (TIF)
Data S7: High resolution fluorescent scan data for the HEG extension data (TIF)
Data S8: High resolution fluorescent scan data for the l-DNA 5′-OH data (TIF)
