# Supplementary material for: Sequence Preference and Initiator Promiscuity for De Novo DNA Synthesis by Terminal Deoxynucleotidyl Transferase
Source: ACS Synth Biol. 2021 Jun 22;10(7):1750–60. doi: 10.1021/acssynbio.1c00142 (PMC8291772; doi:10.1021/acssynbio.1c00142)
Supplement: Supplementary file 1 — sb1c00142_si_001.pdf [file sb1c00142_si_001.pdf]

Supporting information for:

Sequence preference and initiator promiscuity for *de novo* DNA synthesis by terminal deoxynucleotidyl transferase

Erika Schaudy[1], Jory Lietard[1], and Mark M. Somoza[1,2,3]*

[1]Institute of Inorganic Chemistry, Faculty of Chemistry, University of Vienna, Althanstraße 14, 1090 Vienna, Austria

[2]Chair of Food Chemistry and Molecular Sensory Science, Technical University of Munich, Lise-Meitner-Straße 34, 85354 Freising, Germany

[3]Leibniz-Institute for Food Systems Biology at the Technical University of Munich, Lise-Meitner-Straße 34, 85354 Freising, Germany

*Email: mark.somoza@univie.ac.at

The following eight supporting information files are available and linked to this article:

Data S1: Fluorescent intensity data of all experimental data in spreadsheet format. These data were extracted from fluorescence scans of the arrays as described in the methods section.

Data S2: Layout design file with the location and identity of all probes for the HEG arrays. This file contains the sequence, sequence name and x, y coordinates of all HEG sequences on the HEG array. This file can be used with NimbleScan to automatically extract fluorescence intensity data from the scan image of the HEG array (Data S7) as described in the methods section.

Data S3: Layout design file with the location and identity of all probes for the oligonucleotide arrays. This file contains the sequence, sequence name and x, y coordinates of all oligonucleotides sequences on the 2′OMe-RNA, D-DNA 3′-OH, D-DNA 5′-OH and L-DNA 5′-OH arrays. This file can be used with NimbleScan to automatically extract fluorescence intensity data from the scan image of the corresponding array (Data S4, S5, S6 and S8) as described in the Materials and Methods section.

Data S4: High resolution fluorescent scan data for the 2′OMe-RNA data.

Data S5: High resolution fluorescent scan data for the D-DNA 3′-OH extension data

Data S6: High resolution fluorescent scan data for the D-DNA 5′-OH extension data.

Data S7: High resolution fluorescent scan data for the HEG extension data.

Data S8: High resolution fluorescent scan data for the L-DNA 5′-OH data.
