# Supplementary figures and images for: Sequence Preference and Initiator Promiscuity for De Novo DNA Synthesis by Terminal Deoxynucleotidyl Transferase
Source: ACS Synth Biol. 2021 Jun 22;10(7):1750–60. doi: 10.1021/acssynbio.1c00142 (PMC8291772; doi:10.1021/acssynbio.1c00142)

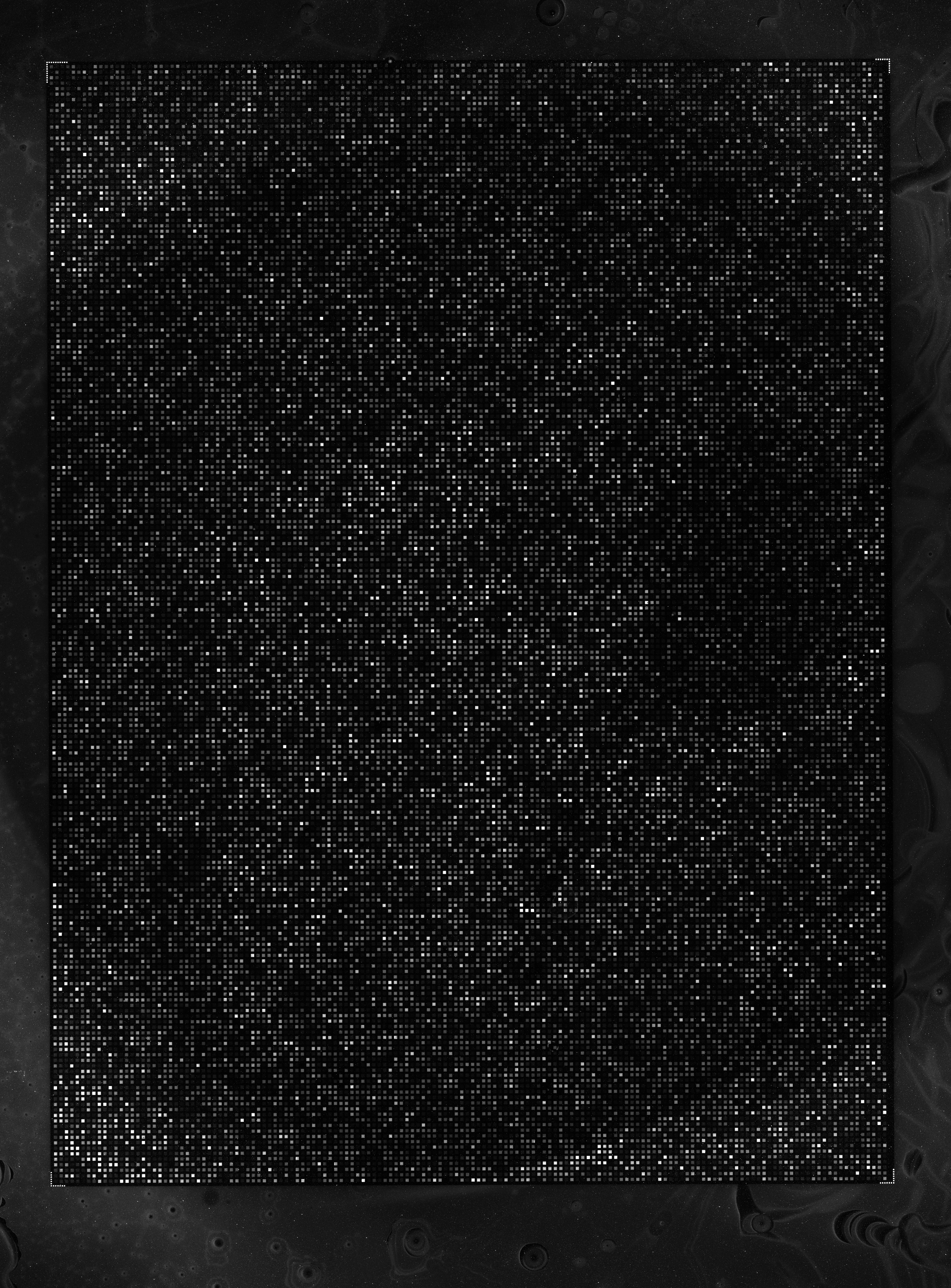

Supplement: Supplementary file 5 — sb1c00142_si_005.tif [file sb1c00142_si_005.tif]

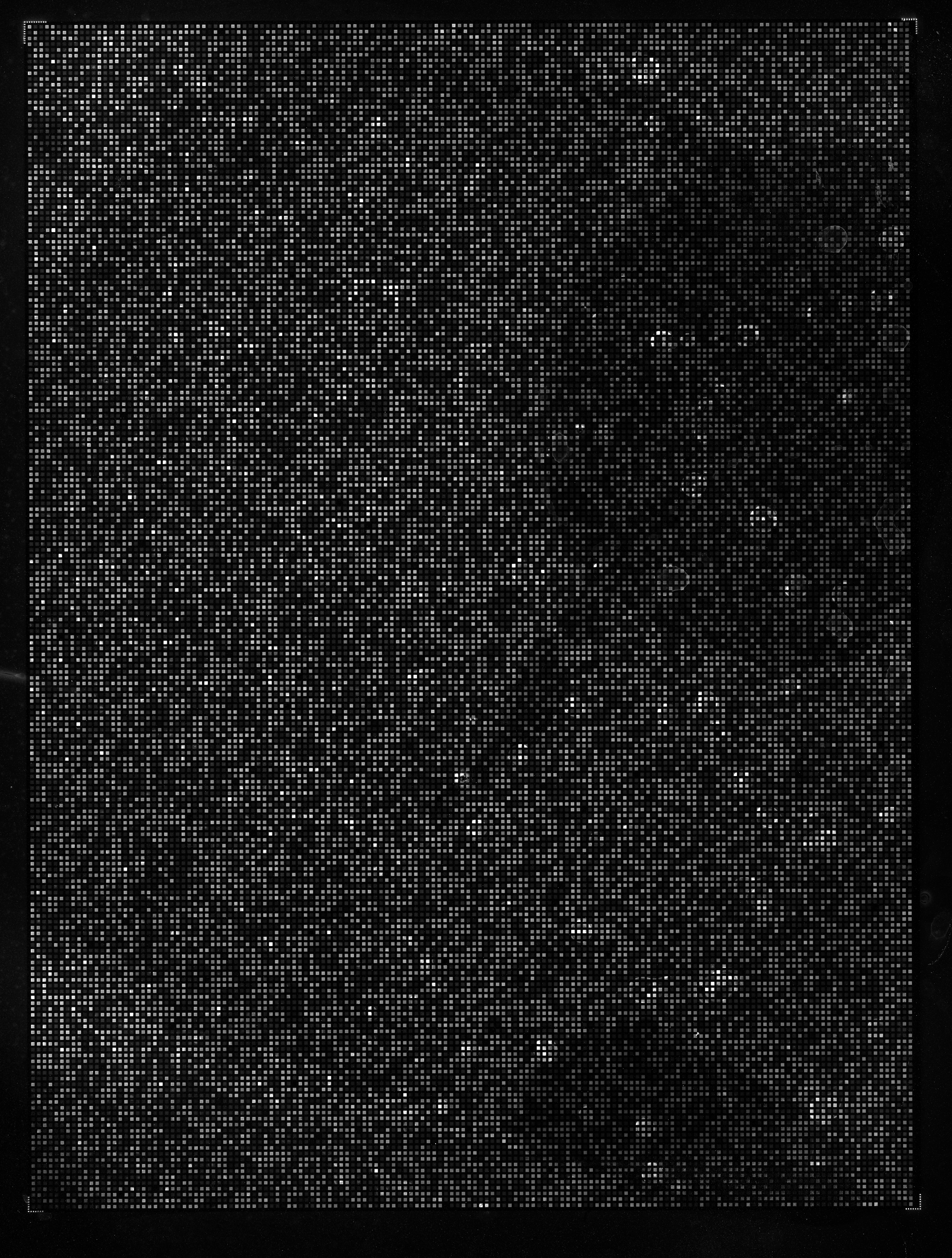

Supplement: Supplementary file 6 — sb1c00142_si_006.tif [file sb1c00142_si_006.tif]

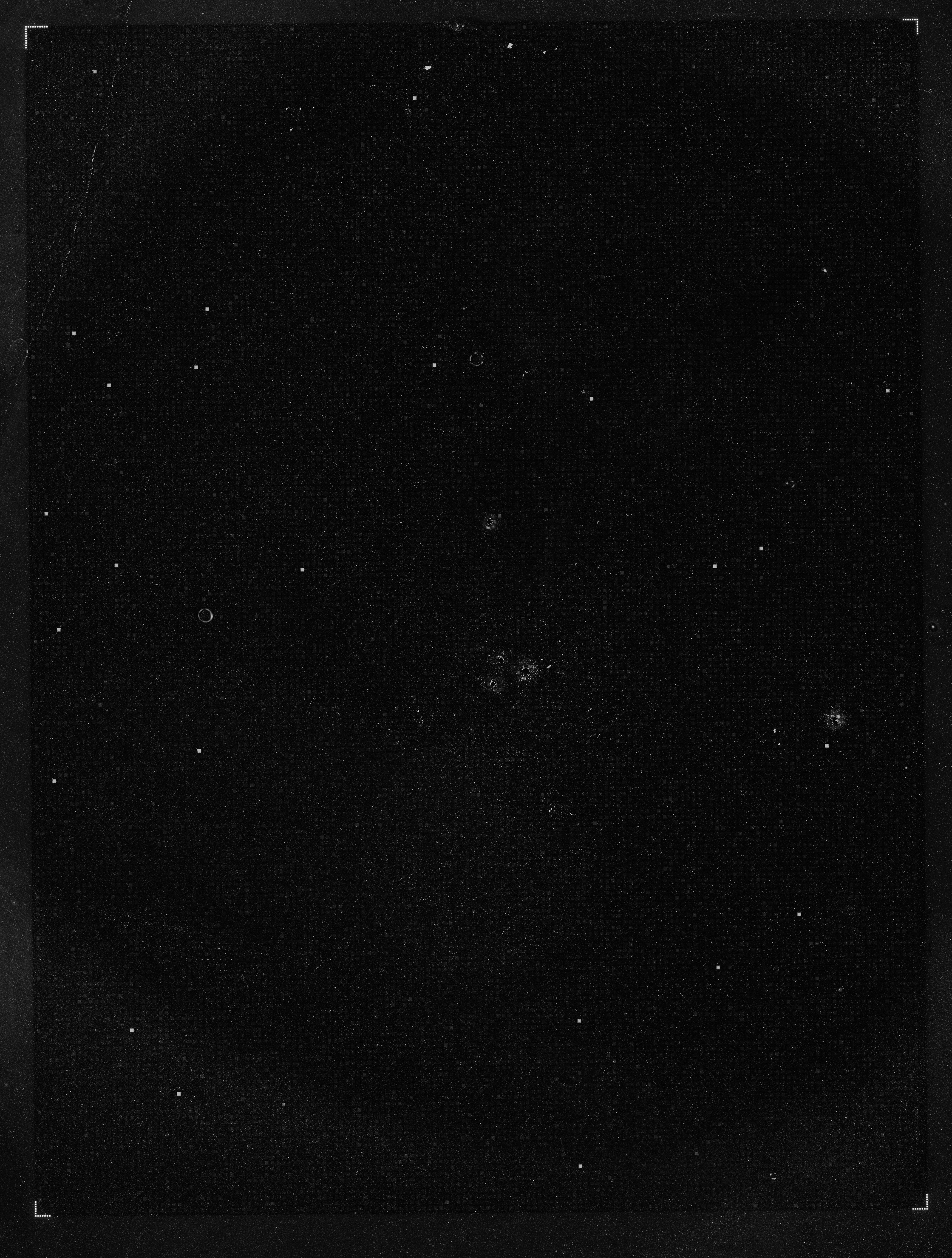

Supplement: Supplementary file 7 — sb1c00142_si_007.tif [file sb1c00142_si_007.tif]

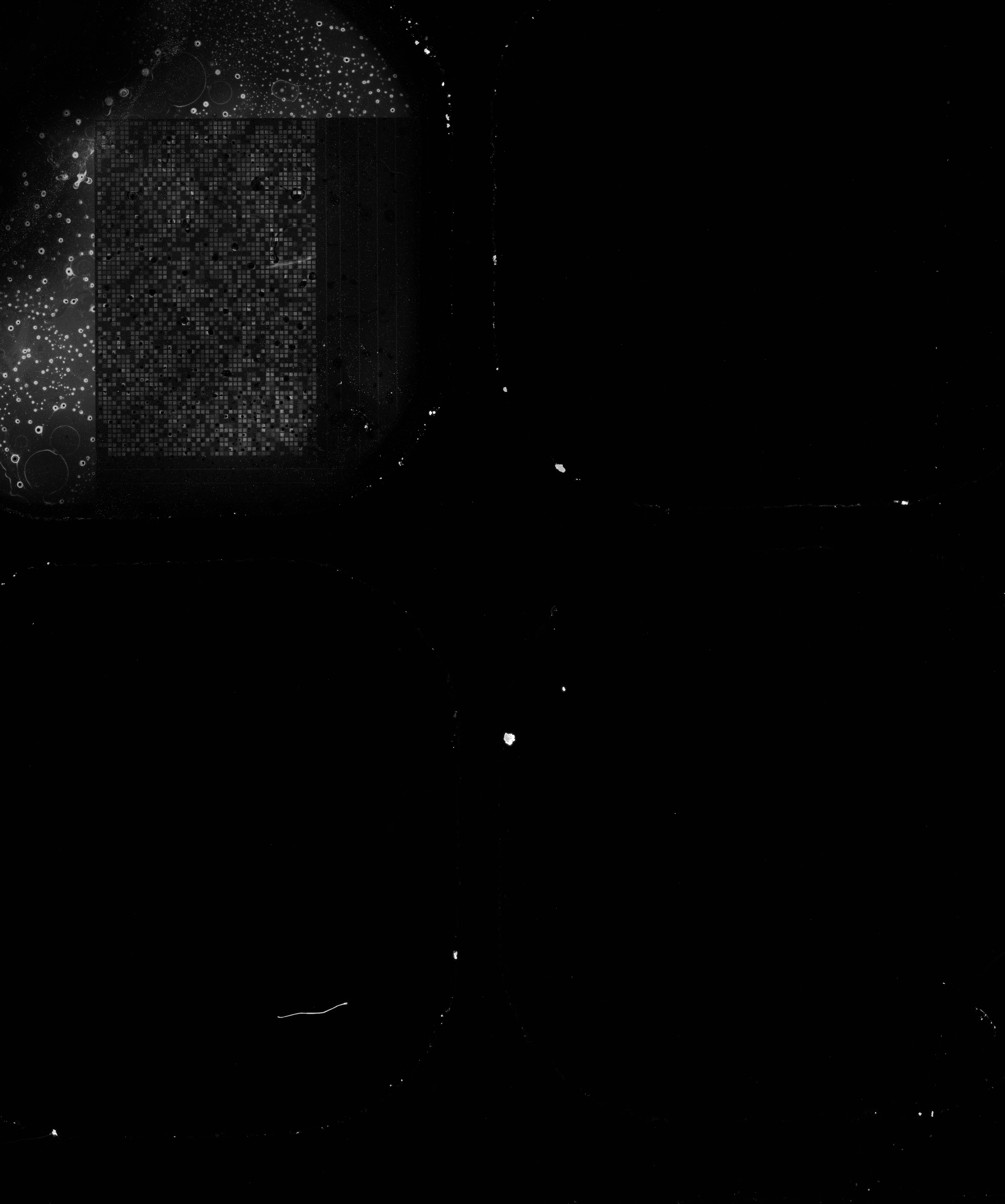

Supplement: Supplementary file 8 — sb1c00142_si_008.tif [file sb1c00142_si_008.tif]

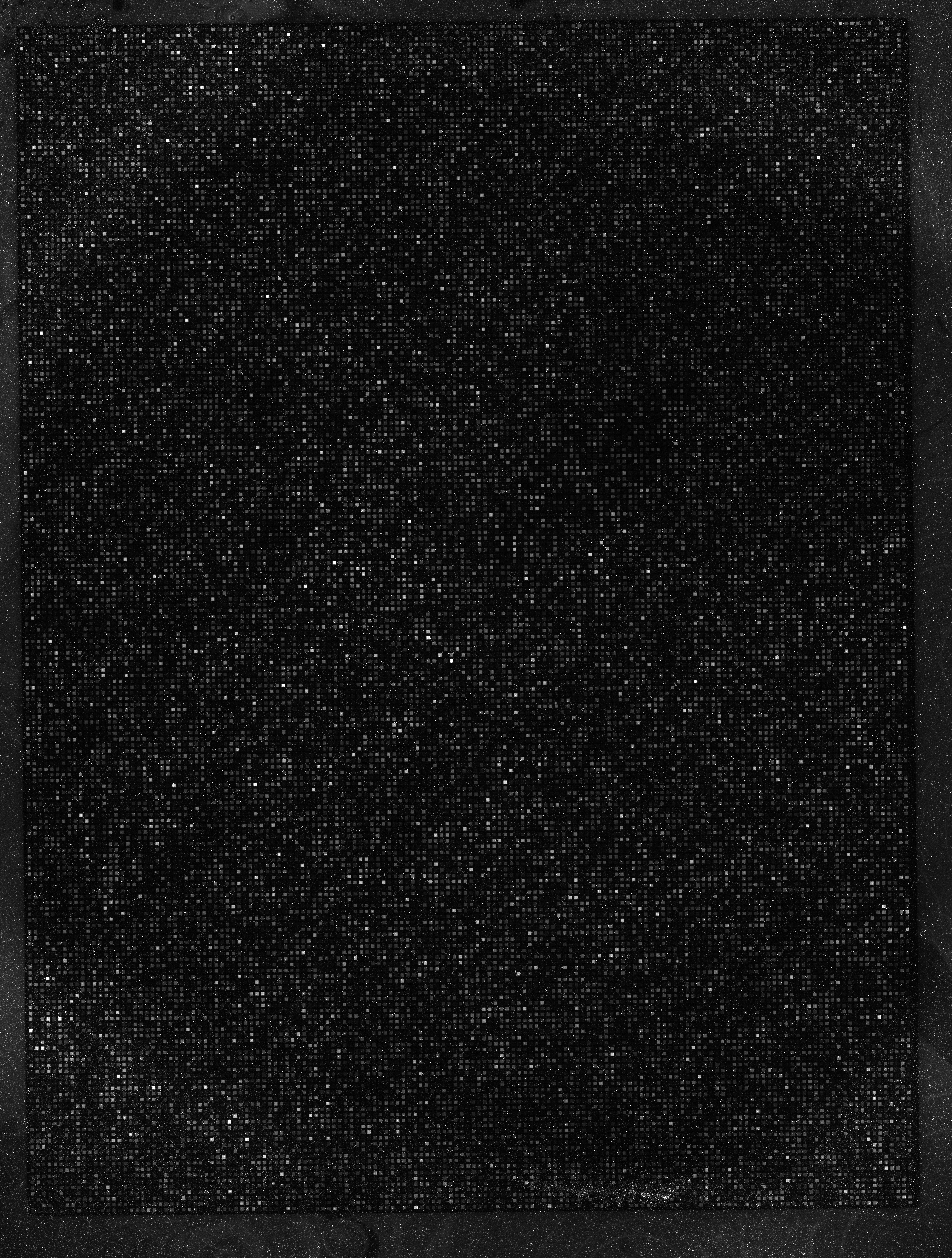

Supplement: Supplementary file 9 — sb1c00142_si_009.tif [file sb1c00142_si_009.tif]
